# Defective Acetylcholine Receptor Subunit Switch Precedes Atrophy of Slow-Twitch Skeletal Muscle Fibers Lacking ERK1/2 Kinases in Soleus Muscle

**DOI:** 10.1038/srep38745

**Published:** 2016-12-09

**Authors:** Shuo Wang, Bonnie Seaberg, Ximena Paez-Colasante, Mendell Rimer

**Affiliations:** 1 Department of Neuroscience and Experimental Therapeutics, Texas A&M University Health Science Center, Bryan, Texas, USA

## Abstract

To test the role of extracellular-signal regulated kinases 1 and 2 (ERK1/2) in slow-twitch, type 1 skeletal muscle fibers, we studied the soleus muscle in mice genetically deficient for myofiber ERK1/2. Young adult mutant soleus was drastically wasted, with highly atrophied type 1 fibers, denervation at most synaptic sites, induction of “fetal” acetylcholine receptor gamma subunit (AChRγ), reduction of “adult” AChRε, and impaired mitochondrial biogenesis and function. In weanlings, fiber morphology and mitochondrial markers were mostly normal, yet AChRγ upregulation and AChRε downregulation were observed. Synaptic sites with fetal AChRs in weanling muscle were ~3% in control and ~40% in mutants, with most of the latter on type 1 fibers. These results suggest that: (1) ERK1/2 are critical for slow-twitch fiber growth; (2) a defective γ/ε-AChR subunit switch, preferentially at synapses on slow fibers, precedes wasting of mutant soleus; (3) denervation is likely to drive this wasting, and (4) the neuromuscular synapse is a primary subcellular target for muscle ERK1/2 function *in vivo*.

Developmental switches in the subunit composition of ligand-gated ion channels that serve as neurotransmitter receptors at glutamatergic, gabaergic, and cholinergic synapses are important for structural and functional synaptic maturation throughout the nervous system. The subunit composition of acetylcholine receptors (AChRs) in the postsynaptic apparatus at developing neuromuscular junctions (NMJ) in the mammalian embryo is α2βγδ. As the synapses mature neonatally, these “fetal” AChRs are gradually replaced by “adult” receptors composed of α2βδε, which have different channel conductance properties^1^,^2^. Germline deletion of *Chrne*^3^,^4^,^5^, the gene encoding AChRε, yields mice that survive embryonic development but harbor NMJs with much lower AChR density that retain AChRγ. These animals have defective neuromuscular transmission, display gradual muscle weakness and atrophy, and die by 2–3 months after birth. Thus the γ/ε-AChR subunit switch is essential for normal skeletal muscle development.

Extracellular signal-regulated kinases 1 and 2 (ERK1/2), the prototypical mitogen-activated protein kinases, mediate a multitude of responses to growth factors and cytokines in cellular proliferation, differentiation, senescence, apoptosis, and survival^6^. ERK1/2 have been implicated in the maintenance of adult skeletal muscle mass^7^ and, seemingly paradoxically, in the control of both the fast-twitch (type 2)^8^ and the slow-twitch (type 1)^9^ fiber type phenotypes. Previously^10^, we generated mice genetically deficient in myofiber ERK1/2. These animals survived development but displayed stunted postnatal growth, muscle weakness and shorter lifespan. We studied two fast-twitch muscles in young adult mice, the sternomastoid (STN) and the tibialis anterior (TA), and found that in both mutant muscles NMJs became fragmented and had reduced AChRε expression. In STN, but not TA, we documented morphological and molecular evidence of partial denervation (e.g. terminal axonal sprouting and induction of the fetal AChRγ-subunit mRNA, respectively). Both muscles also displayed a mixture of fiber loss and mild atrophy, but minimal changes in fiber-type composition. These results were consistent with a role for ERK1/2 in the maintenance of muscle mass, but not of the fast-twitch fiber phenotype, and demonstrated an important role for ERK1/2 in keeping the structural integrity of the mature NMJ *in vivo*. In our previous experiments^10^, we did not study the effects of lack of ERK1/2 on predominantly slow-twitch muscles, nor could we discern whether the phenotypes observed were primarily derived from synaptic or extrasynaptic functions of myofiber ERK1/2.

Here we focused our studies mainly on the soleus (SOL), a prototypical slow-twitch muscle, which unlike the STN and TA has abundant type 1 fibers, rich in mitochondria and highly dependent on oxidative metabolism^11^,^12^. ERKs are most active in type 1 fibers^9^. Thus it is important to determine the consequences that develop for these cells when these kinases are absent. We found that fiber morphology in mutant SOL in young adults (9–14 weeks of age) was much more affected than in either the STN or TA. Type 1 fibers in the SOL, in particular, were very atrophied at this age. We also found fragmented NMJs with low levels of AChR expression and evidence of extensive denervation in these muscles. Moreover, defective mitochondrial function and/or biogenesis were part of the phenotype in mutant SOL in young adults. The striking severity and rapidity in the development of this phenotype facilitated the examination of muscles at different postnatal times. While, at three weeks after birth, control and mutant muscles were similar in fiber morphology, we found evidence of nascent denervation and a defective γ/ε-AChR subunit switch predominantly at NMJs on type 1 fibers. This suggests synaptic instability precedes extrasynaptic changes in myofibers lacking ERK1/2 in SOL. These results also support the notion that the synapse is a primary subcellular target for muscle ERK1/2 function *in vivo*.

## Results

**Figure 1:**
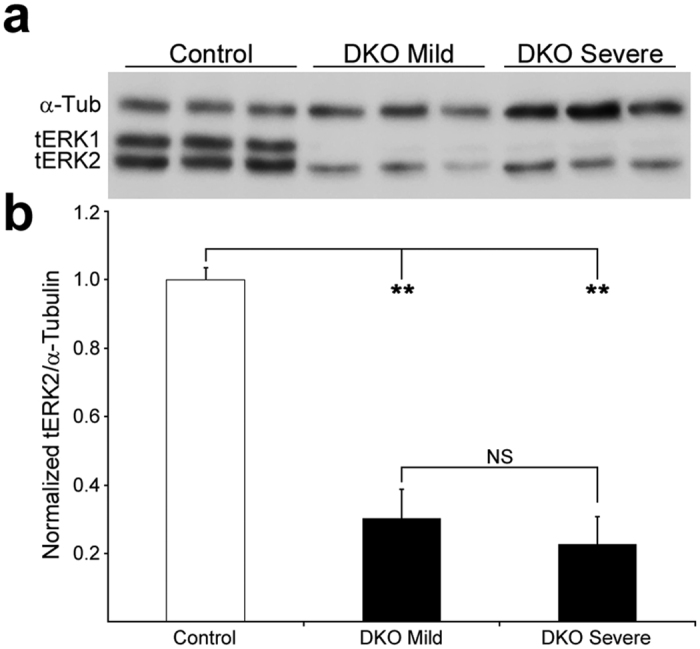
ERK1/2 levels in control and DKO 9 week-old SOL. (**a**) Lysates from 3 separate SOL muscles from control, DKO mild and DKO severe were probed simultaneously for total ERK1/2 (tERK1/2) and for α-tubulin (α-Tub) to check for loading differences. In DKO muscle, ERK1 was completely eliminated and ERK2 was sharply reduced. (**b**) Normalization to α-Tub showed ERK2 levels were 0.30 ± 0.08 –fold and 0.23 ± 0.08 –fold control levels in DKO mild mice and in DKO severe mice, respectively. Values are mean + SD. **p < 0.01; NS, no significant.

We combined a germ line *Erk1* mutation with conditional Cre-loxP *Erk2* inactivation in skeletal muscle to produce mice lacking both ERK1/2 selectively in skeletal myofibers (hereafter DKO mice). Cre was driven by the human α-skeletal muscle actin (*Hsa*) promoter, which is expressed only in myofibers, and not in other cells in the muscle tissue, starting around embryonic day 9.5^13^,^14^,^15^. In the fast-twitch STN and TA we measured a ~90% reduction in ERK2 levels in mutants relative to control^10^. In DKO SOL, ERK2 levels were ~70% lower than in control (Fig. 1). In our initial characterization of these animals, we found they could be divided into two groups according to how fast they lost weight^10^. “Severe” animals lost weight quickly starting at about 7 weeks of age, while “mild” double mutants were able to keep their weight at that age but clearly failed to keep up with controls. Severe and mild DKO mice were generated at about 1:1 ratio, and displayed similar signs of muscle weakness (e.g. forelimb grip strength normalized to body weight) between 7–11 weeks of age^10^. The difference in weight progression between mild and severe DKO mice seemed unexplained by lower ERK2 in the latter, as residual ERK2 levels were similar between mild and severe DKO muscle (p = 0.32; Fig. 1). We do not know the reason for these mild/severe differences but we propose they could be related to undetermined differences in genetic background. For the experiments below all the mutant mice that were 9 weeks or older were DKO mild, while those younger could not be distinguished as mild or severe before that age.

### Effects on Fiber Number, Fiber Type Composition, and Preferential Atrophy of Type 1 Fibers

**Figure 2:**
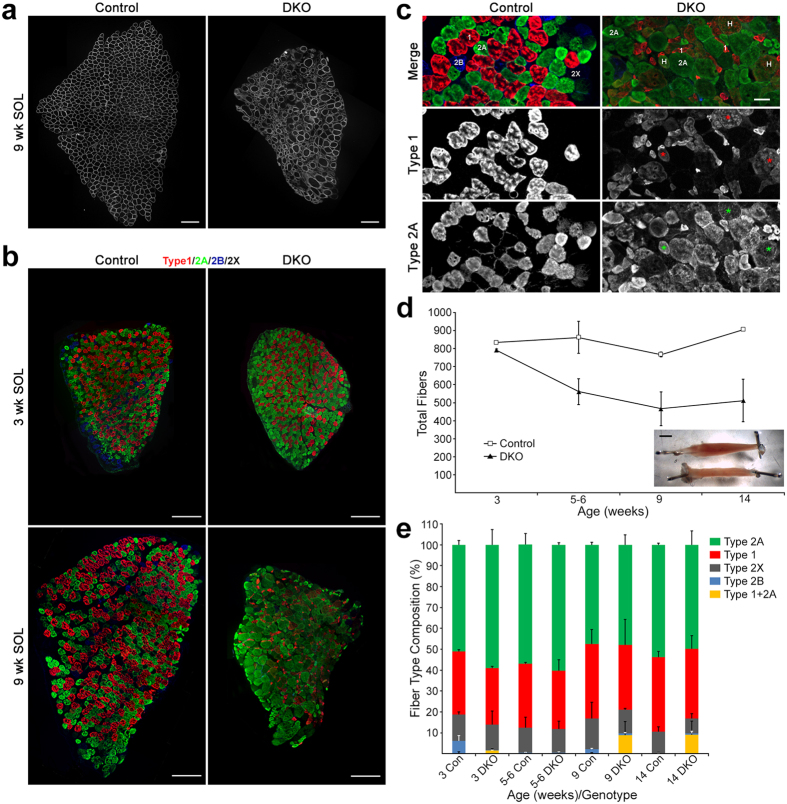
Dramatic wasting of the young adult DKO SOL. (**a**) Cross sections of SOL from 9-week-old control and DKO females stained for dystrophin. Notice reduction in muscle cross-sectional area and dramatic heterogeneity of fiber size in the DKO SOL. Scale bar: 200 μm. (**b**) Simultaneous immunostaining for MyHC types in SOL. Types 1, 2A, and 2B were positively stained, while type 2X was identified by the absence of staining. At 3 weeks, control and DKO SOL were similar in cross sectional area. Note small size of most type 1 fibers in 9 week DKO. Scale bar: 200 μm. (**c**) Higher magnification field for MyHC staining at 9 weeks. Examples of hybrid (H, 1 + 2A) fibers are highlighted with asterisks (*). Scale bar: 50 μm. (**d**) Fiber numbers per cross section in control and DKO SOL at different postnatal times. N = 2–3 muscles per time point/genotype. Values are mean ± SD. Inset shows control (top) and DKO (bottom) representative muscles at 14-weeks (females). Scale bar: 2 mm. (**e**) Fiber type composition in control (CON) and DKO SOL at different postnatal times. N = 2–3 muscles per time point/genotype. Values are mean + SD.

We cut frozen cross sections of SOL muscles from young adults (9–14 weeks old), and stained for dystrophin to mark the cell surface of the myofibers. While control SOL showed a homogenous distribution of fiber sizes, SOL muscle from DKO mice showed a clear reduction in muscle cross-sectional area suggesting fiber loss, with many atrophied and hypertrophied fibers among the remaining myofibers (Fig. 2a). Although we detected fiber loss in DKO STN, such dramatic heterogeneity in fiber size was not observed in DKO STN or TA of the same age^10^. Next, we used immunostaining for the four canonical adult myosin heavy chains (MyHC types 1, 2A, 2B and 2X)^11^ at different postnatal times: 3 (weaning), 5–6, 9, and 14 weeks of age. We followed a protocol that allowed simultaneous staining for all isoforms on the same section; types 1, 2A and 2B by positive staining, 2X by the absence of any staining^16^. Muscles in DKO weanlings appeared similar to controls in cross-sectional area and variability of fiber size (Supplementary Fig. 1), while in young adult DKO, type 1 fibers, in particular, were very atrophied (Fig. 2b,c). We then determined fiber numbers and fiber type composition in controls and DKOs from such stained cross sections. DKO SOL had slightly fewer fibers than control at weaning, but by 5 weeks of age, DKO SOL had many fewer fibers than control, reaching a ~40% reduction relative to control by 9 weeks (Fig. 2d). In 9–14 week-old young adults, controls had 835.8 ± 40.2 fibers on average (n = 4), while DKOs had 493.8 ± 44.3 fibers (n = 5). This was a statistically significant reduction (p = 0.0007; t-test). These data suggested a clear postnatal loss of fibers in the DKO SOL. The inset in Fig. 2d shows representative muscles for control and DKO at 14 weeks and illustrates the extent of wasting at the whole muscle level. Muscle wet weight was consistent with the above data as 14 week-old DKO SOL weighed ~40% less than control (DKO: 9.45 ± 1.21 mg; n = 4 muscles. Control: 14.72 ± 0.88 mg; n = 6 muscles; p = 0.007). The loss in DKO SOL mass was not simply a reflection of the loss of body weight^10^, as even after normalization to body weight, muscle weights remained lower in mutants than in controls (DKO: 0.44 ± 0.07 mg/g, n = 2 animals. Control: 0.58 ± 0.05 mg/g, n = 3 animals).

**Figure 3:**
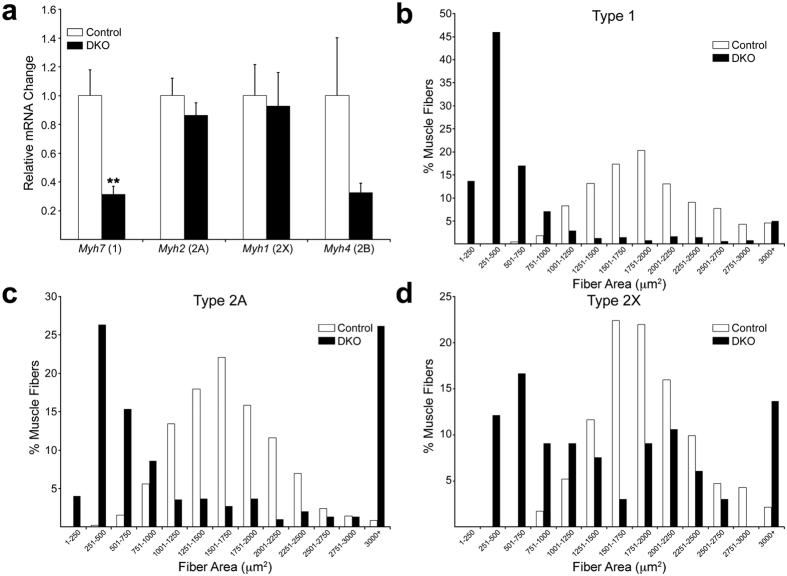
Relative *Myh* mRNA expression and area distribution by fiber type. (**a**) Analysis of real-time PCR for MyHC genes at 9 weeks. N = 6 per genotype. Values are mean + SEM. **p < 0.01, t-test v. control. (**b**,**c**,**d**) Fiber area data for 14-week-old animals were grouped in 250 μm^2^ bins along the X axis and the percentages of fibers in those bins were plotted on the Y axis. In the DKO SOL, type 1 fibers atrophied, while types 2A and 2X both atrophied and hypertrophied. N = 2, control muscles; 3, DKO muscles. Type 1 fibers scored: 675 control, 476 DKO. Type 2A fibers scored: 914 control, 592 DKO. Type 2X fibers scored: 232 control, 66 DKO. Distributions were compared statistically using the Wilconox rank sum test. P < 3.2 × 10^−5^ control v. DKO.

Interestingly, fiber type composition remained largely unchanged, with relative percentages of the most abundant fiber types (1, 2A and 2X) in mutant muscle being similar to those in age-matched control (Fig. 2e). Nevertheless, some changes in fiber composition that affected rare fiber types in the SOL were observed in DKO muscle. Thus, type 2B fibers, which were a very variable ~4% in control at 3 weeks, were absent in DKO at this age. However, type 2B fibers in control SOL reached the same very low levels in DKO by 5–6 weeks (Fig. 2e). Type1 + 2A hybrid fibers (Fig. 2c), which were less than ~0.2% in control, were present at ~5% at 9 weeks or later (Fig. 2e). When fiber counts were sorted by fiber type (Supplementary Fig. 2), type 1, 2A and 2X fibers in young adult (9–14 week-old) DKO were all reduced relative to age-matched control. Thus, cell loss affected all major fiber types and started after 3 weeks of age. At 9 weeks, we also analyzed fiber type composition by measuring mRNA levels for the genes encoding the myosin heavy chains (*Myh1, Myh2, Myh4, Myh7*)^11^ by real-time PCR. In DKO SOL, a statistically significant ~3-fold reduction of *Myh7* (type 1) mRNA (p = 0.004), and a tendency toward reduction in *Myh4* (type 2B) transcript (Fig. 3a) were observed. In the fast-twitch DKO STN and TA muscles, no statistically significant changes in expression of myosin heavy chain genes were detected, even though a tendency toward decrease was seen for *Myh7* (Supplementary Fig. 3). Our results are better interpreted in the context of the normal levels of *Myh7* expression in each of the muscle groups. *Myh7* expression in control SOL was ~30-fold higher than in STN and TA (Supplementary Fig. 3). Thus, because of higher control levels, the 70% reduction in *Myh7* mRNA expression in DKO SOL is much more meaningful than a similar tendency in TA or STN, where *Myh7* expression is normally very low (i.e. there are very few type 1 fibers in these muscles). The reduction in *Myh7* mRNA levels in the DKO SOL was associated with the preferential atrophy of these fibers (Fig. 2b,c) and not with fiber switching, as relative fiber type composition was largely similar between control and DKO SOL (Fig. 2e). Indeed, a histogram of fiber areas showed that ~70% of type 1 fibers in 14 week DKO SOL were smaller than 750 μm^2^, while almost no fibers that small were found in controls (Fig. 3b). On the other hand, very small (<750 μm^2^) and very large (>3000 μm^2^) 2A and 2X fibers were much more abundant in DKO SOL than in control (Fig. 3c,d), suggesting that these fast-twitch fibers undergo both atrophy and hypertrophy. At 5–6 weeks, average area for all major fiber types was statistically larger in DKO SOL than in control (Supplementary Fig. 4). This result suggests that fibers in DKO SOL may hypertrophy before atrophy ensues, perhaps as a compensation for fiber loss.

We also examined type 1 fiber area in two fast-twitch muscles, STN and extensor digitorum longus (EDL) (Supplementary Fig. 5). Type 1 fibers are present at very low numbers in these muscles. Atrophy of type 1 fibers was evident in the 14-week DKO STN as fibers >300 μm^2^ in area were absent, while present in control. In 14-week DKO EDL, type 1 fiber atrophy was less robust yet statistically present as average fiber area was ~25% lower than control (DKO: 132.17 ± 8.87 μm^2^, n = 51 fibers, 4 mice. Control: 178.50 ± 12.18 μm^2^, n = 42 fibers, 4 mice; p = 0.004, t-test; p = 0.008, Wilconox rank sum test). Thus, atrophy of type 1 fibers occurred in all muscles studied.

### Effects on Synapse Morphology and Denervation-Related Molecular Markers

**Figure 4:**
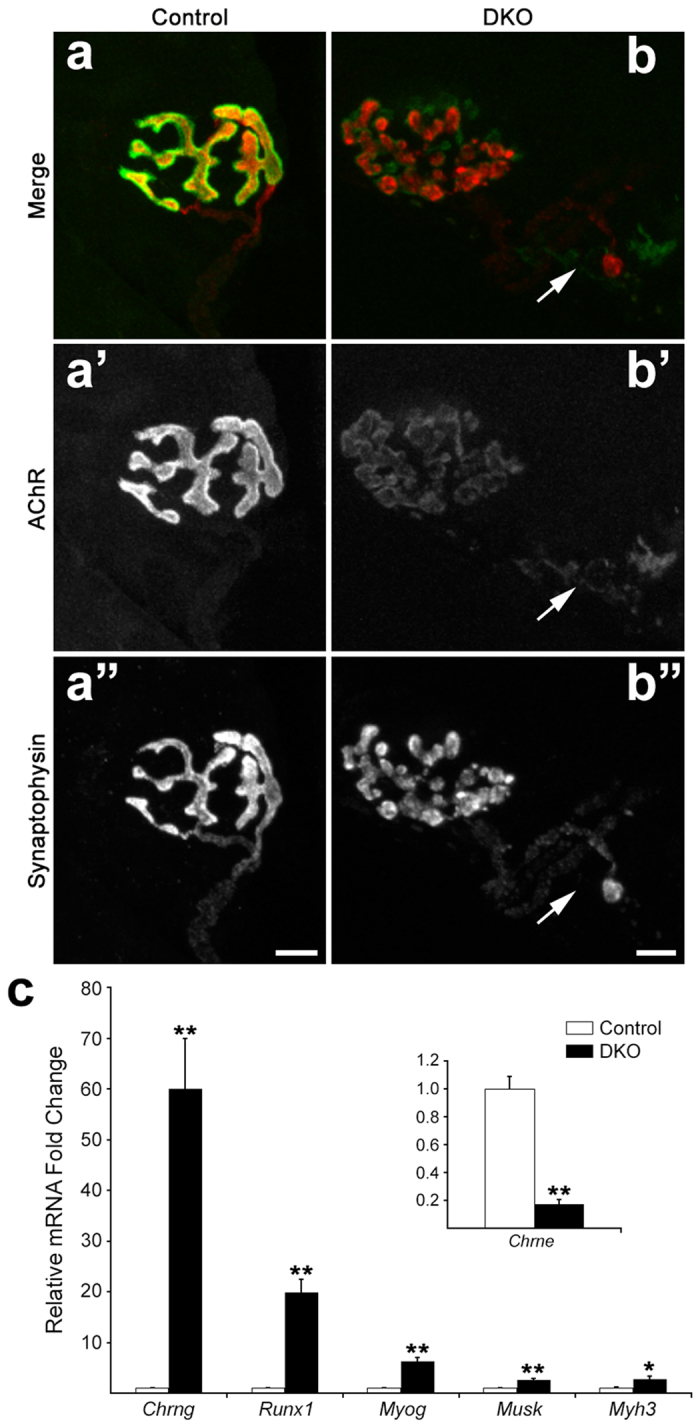
Synaptic alterations in young adult DKO SOL. (**a**) Confocal “en face” view of a 9-week control NMJ labeled with fluorescein-BTX to mark AChRs (a’) and with antibodies to synaptophysin, followed by rhodamine-conjugated secondary antibodies, to label nerve terminals (a”). Long, continuous AChR domains are tightly apposed by nerve terminals. (**b**) Confocal “en face” view of a fragmented NMJ in 9-week DKO muscle with small, mostly round, dim AChR domains (b’) variably apposed by nerve terminal staining (b”). Arrow: Side view of an aneural synaptic site with very weak AChR staining and axonal retraction bulb. Scale bar: 10 μm. (**c**) Real-time PCR of mRNA for denervation and postsynaptic genes at 9 weeks. *Chrne* was reduced by ~5-fold (inset), while the other mRNAs were increased between 3- and 60-fold. N = 6 per genotype. Values are mean + SEM. **p < 0.01; *p < 0.05; t-test v. control.

As in STN and TA^10^, NMJs with signs of fragmentation and diminished AChR expression could be found in young adult DKO SOL (Fig. 4a,b). Using real-time PCR, we found a ~5-fold reduction in AChRε mRNA in DKO SOL relative to control (inset Fig. 4c; p = 0.000007). There was morphological and molecular evidence of partial denervation in the DKO SOL in young adults (Fig. 4). Most notably, there was a ~60-fold increase in mRNA for *Chrng*, the gene encoding the distinctive γ-subunit of the fetal AChR^17^ (p = 0.00014), and a ~20-fold induction for *Runx1* mRNA (p = 0.00004), a transcription factor highly induced in skeletal muscle after denervation^18^,^19^. Furthermore, the myogenic factor myogenin (*Myog*), the embryonic myosin isoform (*Myh3)*, and the agrin-transducing receptor *Musk*, all known markers of denervation^19^,^20^,^21^, were also elevated between ~3- and 6-fold over control (p = 0.00003, 0.013, 0.0004, respectively) (Fig. 4c).

**Figure 5:**
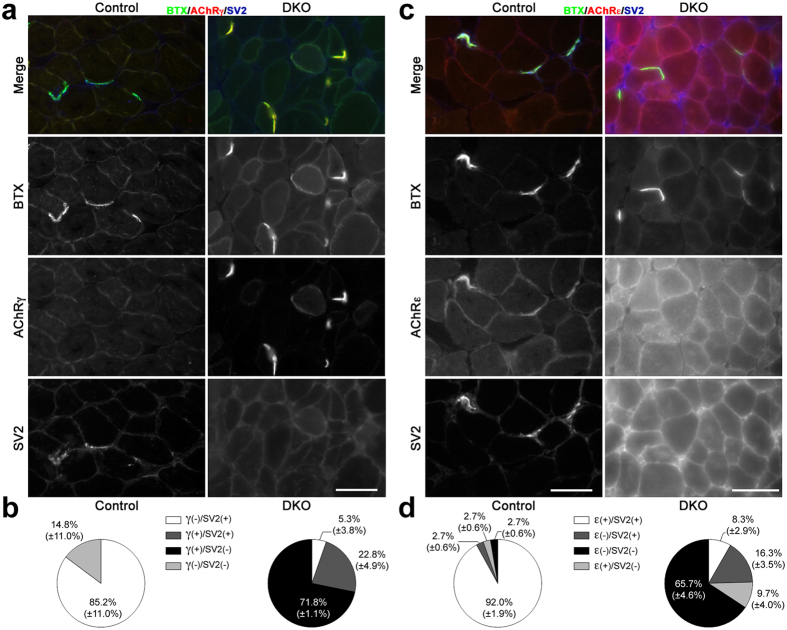
AChRγ/ε staining in 14 week SOL cross sections. (**a**) Control pictures show 3 innervated NMJs (SV2+) that failed to label for AChRγ (AChRγ-). DKO pictures show 5 AChRγ+/SV2- synaptic sites. Scale bar: 50 μm. (**b**) Quantification for AChRγ/SV2/BTX staining. Values are mean ± (SD). N = 2 control muscles, 74 endplates; 3 DKO muscles, 100 endplates. (**c**) Control pictures show 3 innervated NMJs (SV2+) that label for AChRε (AChRε+). DKO pictures show 5 synaptic sites, two strongly positive for BTX and 3 with weak BTX staining. All 5 sites lack both AChRε and SV2 staining. Scale bars: 50 μm. (**d**) Quantification for AChRε/SV2/BTX staining. Values are mean ± (SD). N = 2 control muscles, 77 endplates; 3 DKO muscles, 77 endplates.

Denervation in the adult induces replacement of the AChRε subunit with the AChRγ subunit at synaptic sites^22^. Staining for AChRγ with subunit-specific antibodies^23^ on cross sections of 14 week-old muscle showed that ~90% of BTX-labeled synaptic sites incorporated these fetal receptors in DKO SOL (Fig. 5). Absence of co-staining for the presynaptic marker SV2 suggested denervation at ~70% of these sites (Fig. 5). Even most of the SV2+ NMJs were also AChRγ+ (Fig. 5b). Staining of serial sections with AChRε subunit-specific antibodies^23^ was consistent with the AChRγ staining, and directly showed that only ~10% of synaptic sites were AChRε+/SV2+ in DKO SOL while over 90% were so in control (Fig. 5b).

Thus, by 14 weeks in DKO SOL, most NMJs appeared denervated and had γ-subunit-containing AChRs. The presence of type 1 + 2A hybrid fibers in DKO SOL of young adults (Fig. 2e, above), and the selective reduction of *Myh7* mRNA (Fig. 3a) were also consistent with denervation/re-innervation cycles occurring in this muscle^24^,^25^,^26^. Thus, DKO SOL in young adults showed signs of marked NMJ instability that included reduction of AChRε expression and loss of nerve-muscle contact.

### Mitochondrial Alterations

**Figure 6:**
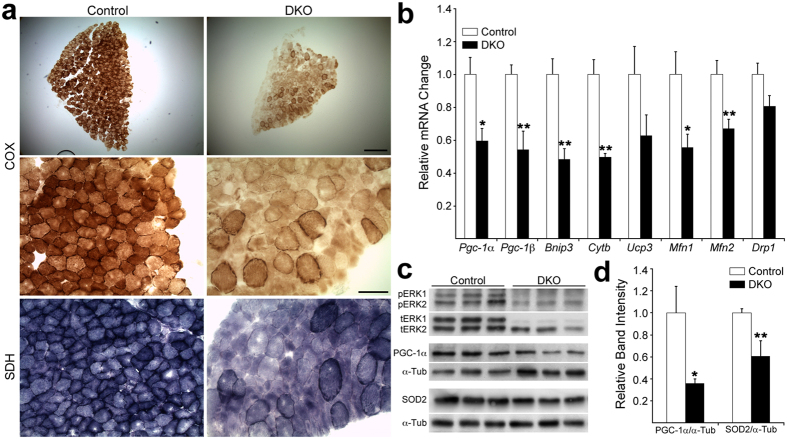
Mitochondrial phenotype in young adult DKO SOL. (**a**) Reduced cytochrome oxidase (COX) and succinate dehydrogenase (SDH) activity on control and DKO SOL cross sections of 9 week mice. Scale bars: Top row pictures 200 μm; middle and bottom row pictures 50 μm. N = 2 per genotype. (**b**) Real-time PCR for mRNA of mitochondrial markers on 9 week SOL. N = 6 per genotype. Values are mean + SEM. *p < 0.05; **p < 0.01; t-test v. control. (**c**) Top 2 Western blots illustrate active (pERK1/2) and total (tERK1/2) ERK protein levels in 9-week control and DKO SOL. The same lysates were probed in separate blots for PGC-1α (middle blot) and SOD2 (bottom blot). Loading of PGC-1α and SOD2 blots was assayed with α-tubulin antibody (α-Tub, bottom blot). (**d**) PCG-1α and SOD2 quantification. N = 3 per genotype. Values are mean + SD. *p < 0.05; **p < 0.01; t-test v. control.

Given that over 70% of myofibers in the SOL are metabolically oxidative (type 1 and type 2A), we assessed oxidative capacity on cross sections of 9–14 week-old SOL by cytochemical staining for cytochrome oxidase (COX) and succinate dehydrogenase (SDH)^27^. These enzymes showed much reduced activity in DKO SOL relative to control at this age (Fig. 6a), consistent with diminished mitochondrial function. Next, we used real-time PCR to measure mRNA levels of the regulators of mitochondrial biogenesis peroxisome proliferator-activated receptor γ coactivator 1α (*PGC-1α*^28^) and *PGC-1β*^29^, the mitochondrial fusion/fission markers mitofusin 1 and 2 (*Mfn1, Mfn2*), dynamin-related protein 1 (*Drp1*)^30^, and the mitochondrial markers BCL2/Adenovirus E1B 19 kDa interacting protein 3 (*Bnip3*), cytochrome b (*Cytob*), and uncoupling protein 3 (*Ucp3*). While *Ucp3* tended to decrease and *Drp1* did not change, we found statistically significant ~2-fold reductions in mRNA for *PGC-1β, Mfn1* and *Mfn2, Bnip3, Cytob* and *PGC-1*α (p values = 0.005, 0.02, 0.009, 0.001, 0.0003, 0.01, respectively) (Fig. 6b). A ~3-fold reduction in PGC-1α protein was also detected in the DKO SOL (p = 0.01; Fig. 6c). In contrast, *PGC-1α* mRNA in the fast twitch STN and TA muscles were not different between mutant and control at the same age (Supplementary Fig. 6). SOD2, a mitochondrial protein that is a direct downstream target of PCG-1α regulation^31^, was reduced ~40% in DKO SOL (p = 0.009; Fig. 6c). Together, these results suggest that absence of muscle ERK1/2 specifically in the SOL leads to reduced mitochondrial biogenesis, which may account, at least in part, for the lower oxidative capacity reflected by the decrease in COX and SDH activities. These mitochondrial alterations were also in keeping with the ample evidence of denervation found in these muscles^26^,^32^.

### Early Events That Precede Fiber Atrophy And Loss

**Figure 7:**
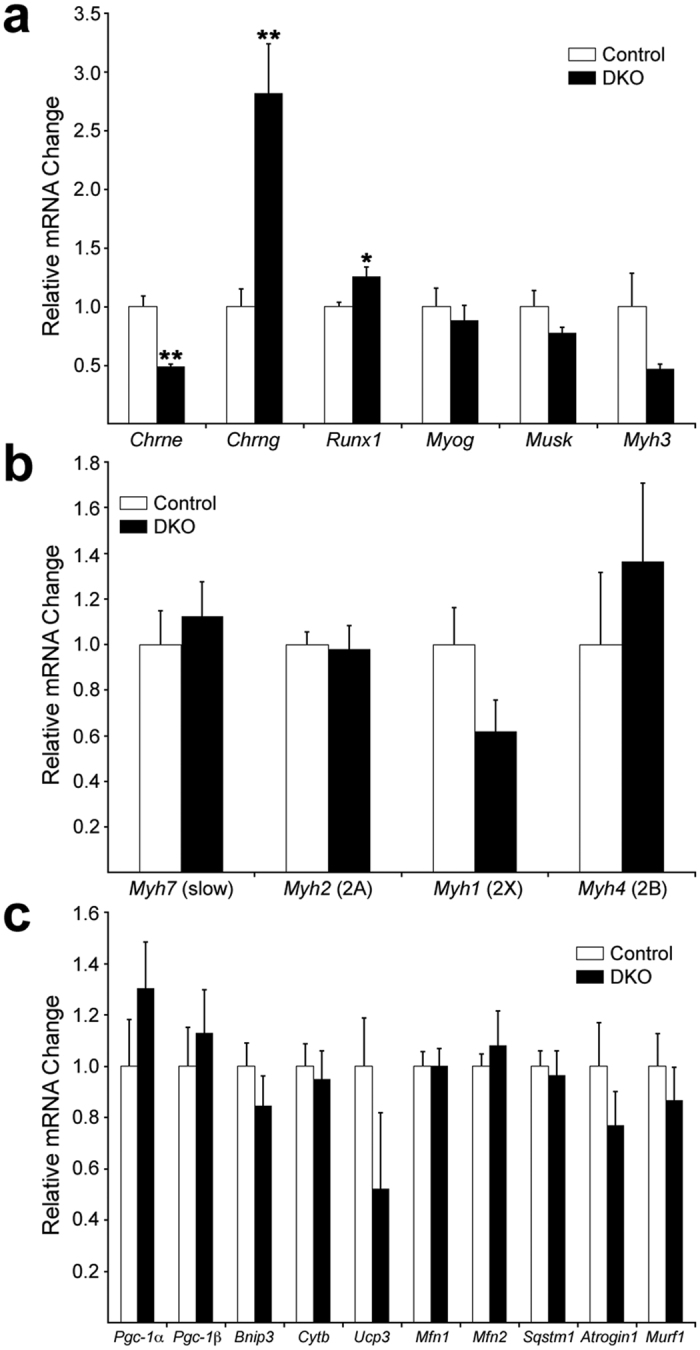
Relative mRNA expression at 3 weeks. (**a**) Real-time PCR of mRNA for denervation and postsynaptic genes at 3 weeks. *Chrne* was reduced by ~2-fold, while *Chrng* and *Runx1* were increased by ~3- and 1.3-fold, respectively. (**b**) Real-time PCR of mRNA for MyHC genes. (**c**) Real-time PCR of mRNA for mitochondrial (*Pgc-1α – Mfn2*) and protein degradation genes (*Sqstm1* (p62), *Atrogin1* and *Murf1*). None of these genes showed any expression changes with genotype at this age. N = 6 control, 5 DKO. Values are mean + SEM. **p < 0.01; *p < 0.05; t-test v. control.

**Figure 8:**
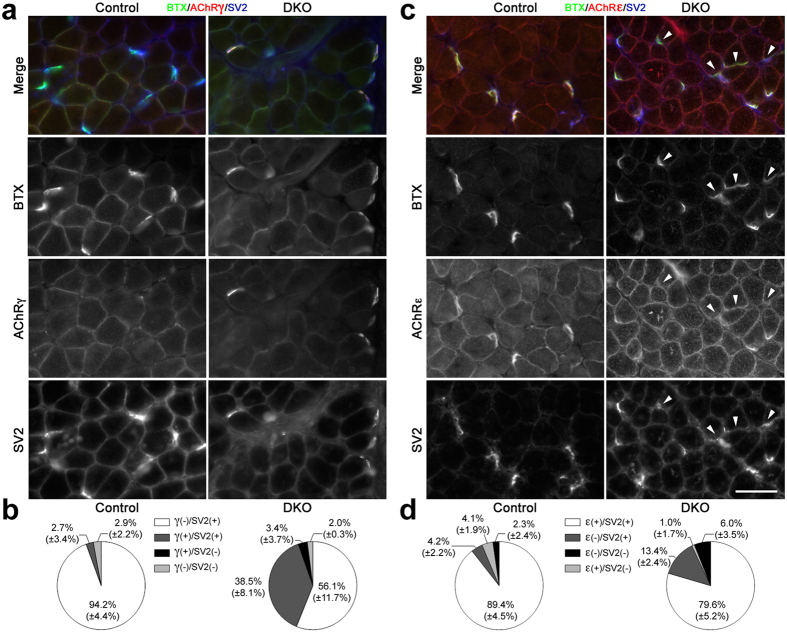
AChRγ/ε staining in 3 week SOL cross sections. (**a**) Control pictures show 7 innervated NMJs (SV2+) that were AChRγ-. DKO pictures show 4 AChRγ+/SV2+ NMJs in focus. Scale bar: 50 μm. (**b**) Quantification for AChRγ/SV2/BTX staining. Values are mean ± (SD). N = 3 control muscles, 139 endplates; 3 DKO muscles, 148 endplates. (**c**) Control pictures show 5 innervated NMJs (SV2+) that label for AChRε (AChRε+). DKO pictures show 4 synaptic sites (arrowheads) where AChRε staining is not above extrasynaptic background levels on same fiber (AChRε-). The other endplates are AChRε+. Scale bar: 50 μm. (**d**) Quantification for AChRε/SV2/BTX staining. Values are mean ± (SD). N = 3 control muscles, 145 endplates; 3 DKO muscles, 109 endplates.

**Figure 9:**
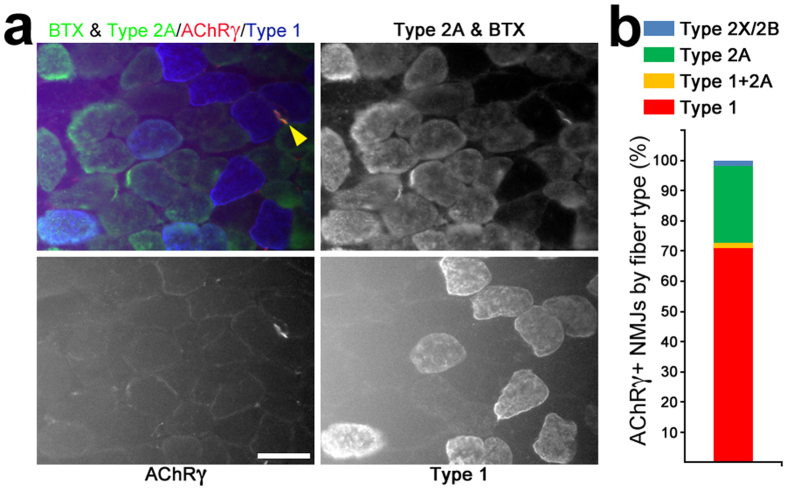
AChRγ staining preferentially at NMJs on type 1 fibers in 3-week-old DKO SOL. (**a**) Four NMJs labeled with fluorescein BTX, three on type 2A fibers and one (yellow arrowhead) on a type 1 fiber. Only the latter is AChRγ+. BTX and type 2A staining were visualized in fluorescein channel; AChRγ visualized in rhodamine channel; type 1 visualized in Cy5 (647 nm) channel. Scale bar: 50 μm. (**b**) Quantification of AChRγ+ NMJs by fiber types in DKO SOL. N = 3 muscles; 56 NMJs.

To search for potential mechanisms that drive the phenotype, we analyzed gene expression at 3 weeks. We chose this time point because fiber loss and atrophy were largely absent (Fig. 2b,d and Supplementary Fig. 1). We specifically focused on genes whose expression levels were altered at 9 weeks: *Myh7* (Fig. 3a), the denervation/synaptic markers (Fig. 4c) and mitochondrial genes (Fig. 6b) as well as key genes for autophagy and ubiquitin-proteasome-mediated proteolysis, since these are primary mechanisms mediating muscle atrophy in most muscle wasting situations^33^,^34^. Only expression of *Chrne* (decreased by ~2-fold; p = 0.0006), *Chrng,* and *Runx1* (increased by ~3- and ~1.3-fold, respectively; p = 0.004 and 0.02, respectively) were statistically altered at this early time (Fig. 7a). Neither *Myh7*, or the other MyHC genes (Fig. 7b), nor the mitochondrial- or muscle atrophy-related genes (Fig. 7c) exhibited differences at the mRNA level between DKO and control SOL at this early age. These results suggested that synaptic alterations that may lead to denervation preceded all other phenotypic changes in DKO SOL. To further support this conclusion, we co-stained for AChRγ, SV2 and BTX to check for the presence of synapses expressing the fetal form of the AChR (Fig. 8). Control muscles at 3 weeks had a very small and variable fraction of AChRγ+/SV2+ synapses (2.7 ± 3.4%), consistent with previous reports that showed that AChRγ persists in some NMJs about a week longer in normal SOL than in fast-twitch muscles^35^. However, in DKO muscle 38.5 ± 8.1% of NMJs were AChRγ+/SV2+ at 3 weeks of age (Fig. 8a,b). This ~10-fold difference in AChRγ+/SV2+ NMJs between DKO and control was statistically significant (p = 0.002). Consistent with the slight but statistically significant increase in *Runx1* mRNA (Fig. 7) and the presence of a few type1 + 2A hybrid fibers in the DKO SOL at 3 weeks (Fig. 2e), a small, highly-variable fraction of endplates (3.4 ± 3.7%) appeared denervated as they lacked SV2 staining and were AChRγ+ (Fig. 8b). AChRγ+/SV2- synaptic sites were not detected in control muscles (Fig. 8b). Staining for AChRε at this early age showed a statistically significant increase in AChRε-/SV2+ NMJs in DKO muscle relative to control (13.4 ± 2.4% and 4.2 ± 2.2%, respectively; p = 0.008) and a corresponding reduction in AChRε+/SV2+ endplates (89.4 ± 4.5% control, 79.6 ± 5.2% DKO) that almost reached statistical significance (p = 0.07) (Fig. 8c and d). Because the fraction of AChRγ+/SV2+ NMJs (38.5 ± 8.1%) is larger than the fraction of AChRε-/SV2+ NMJs (13.4 ± 2.4%), it is likely that many of the AChRε+/SV2+ junctions are also AChRγ+ in weanling DKO muscles. In this context, it should be mentioned that extrasynaptic background staining tended to be higher, particularly in DKO muscle, when staining for AChRε than when staining for AChRγ (Figs 5 and 8), which made the quantification more challenging for the former than for the latter. We next stained simultaneously for AChRγ, positively for MyHCs 1 and 2A, negatively for 2X + 2B, and added BTX to try to determine if AChRγ-containing NMJs preferentially localized to a particular fiber type at this early time in DKO SOL. We found that 71% of AChRγ+ endplates occurred on type 1 fibers (40/56 endplates, n = 3 muscles), while 25% and 2% of endplates containing this subunit were found on type 2A and 2X/2B fibers, respectively (Fig. 9). Thus despite higher proportion of type 2 fibers in SOL (Fig. 2e), synapses on type 1 fibers were much more susceptible to alteration following ERK1/2 deficiency than those on type 2 fibers. Taken together, these results strongly suggest that a defective γ/ε-AChR switch and perhaps even loss of synaptic connectivity, particularly on type 1 fibers, are early events in the development of the wasting phenotype in DKO SOL.

## Discussion

The analysis of the slow-twitch SOL muscles in mice lacking ERK1/2 in myofibers reveals that these kinases critically regulate type 1 fiber size *in vivo*. This regulation may extend to type 1 fibers in many muscle groups as their atrophy was detected in fast-twitch STN and EDL. ERK1/2 also appears to control the γ/ε-AChR switch at maturing NMJs in SOL, whose impairment precedes any other alterations.

ERK phosphorylation levels in adult rat skeletal muscle fibers are sensitive to the firing pattern of the motoneurons that innervate them. In particular, a low frequency/high amount impulse pattern (20 Hz), typical of slow motoneurons that innervate type 1 fibers, elicited the highest increase in ERK phosphorylation when applied to adult rat SOL muscle^9^. Firing patterns more similar to those for type 2B or type 2A motor units failed to stimulate, or more modestly induced ERK activation, respectively^9^. These results predict that removal of ERK from skeletal muscle fibers should more dramatically affect the type 1 muscle fibers. Our results in mouse SOL are consistent with this prediction and support the notion that ERK1/2 play a critical role in type 1 fibers. Although type 2 fibers are also affected as they undergo both atrophy and hypertrophy (Fig. 3), the dramatic myofiber wasting in young adult DKO SOL directly correlates with its higher content of type 1 fibers (~30%, this work and others (e.g.^16^)) relative to the previously examined STN and TA^10^. Type 1 fiber atrophy was also detected in fast twitch muscles. In DKO STN there was a clear absence of large type 1 fibers, while in the EDL the atrophy was less robust but present statistically. The difficulty of detecting robust atrophy in EDL might be related to the already small size of its few slow fibers. Indeed, type 1 fibers in control EDL were on average as small as the most atrophied fibers in the DKO SOL (Fig. 3b). In addition, it is known that type 1 fibers in the rat EDL, for example, are resistant to denervation-induced atrophy, while they are very sensitive in the SOL^24^. Rat type 1 fibers in SOL are ~3 times larger than in the EDL^16^. Thus, it is possible that small type 1 fibers for some reason are much less sensitive to the lack of ERK1/2 than large type 1 fibers.

Since our DKO mice lack ERK1 in all cells, including motoneurons, it is reasonable to ask whether the absence of ERK1 in motoneurons lowers the threshold for the defects in NMJ maturation we observed. This does not appear to be the case as SOL morphology and relative mRNA levels of synaptic/denervation markers in young adult *Erk1*^−/−^ mice were similar to control (Supplementary Fig. 7).

A primary role of myofiber ERK1/2 in DKO SOL seems to be synaptic rather than extrasynaptic since a defective γ/ε-AChR subunit switch, largely confined, but not exclusive to NMJs on type 1 fibers, preceded fiber loss and atrophy. *Chrne*^−/−^ mice survive development and go normally through some milestones of postnatal synaptic maturation such as synapse elimination^3^,^4^. However, their NMJs, which retain the fetal AChRγ subunit, have a much lower density of AChRs and show structural postnatal abnormalities such as fragmentation^4^,^5^. *Chrne*^−/−^ mice also develop muscle atrophy, show a fast-to-slow fiber type transition in both fast- and slow-twitch muscles^36^, and fail to live past 14 weeks. These results indicate that ε-containing AChRs play a structural role in the normal maturation of the NMJ, and that a defective γ/ε-switch leads to alterations in the maintenance of muscle fibers. Thus the defective γ/ε-switch may be a contributor to the striking wasting of the SOL muscle in ERK1/2 deficient mice. A reduction of AChRε expression is common to the STN, TA^10^, and SOL (Fig. 3c) in young adult DKO animals. These reductions are averages for the whole muscle, and it is possible they are larger at some synaptic sites than at others, as suggested by the AChRγ/ε staining (Fig. 4). Specific molecular mechanisms by which ERK1/2 upregulate AChRε expression *in vivo* remain to be determined, although transcriptional regulation is likely one of them^37^,^38^. As in the *Chrne*^−/−^ mice, induction of AChRγ expression in ERK1/2-deficient fibers may arise as compensation to the reduction in AChRε and/or after denervation. Alternatively, ERK1/2 may be necessary to repress AChRγ expression, at least in type 1, and perhaps some type 2A fibers.

There are some differences between the ERK1/2-deficient and *Chrne*^−/−^ SOL that suggest that processes other than the defective γ/ε-AChR switch also contribute to the phenotype of the former. Neither fiber loss, in general, nor atrophy of type 1 fibers or reduction of *Myh7* expression, in particular, were reported in *Chrne*^−/−^ SOL at least up to 9 weeks of age^36^. Moreover, although physiological evidence^3^ and presence of terminal axonal sprouts^4^ suggested some degree of paralysis or partial denervation, it is unclear if anatomical denervation actually occurs at AChRε-less NMJs. One could speculate that had the *Chrne*^−/−^ mice survived longer than the 2–3 months of their average lifespan, clearer signs of denervation might have been detected in their SOL. In DKO SOL, denervation extended to ~70% of synaptic sites by 14 weeks of age (Fig. 5). This denervation is myogenic, and not neurogenic, in origin, as no changes in activate or total ERK2 levels were detected in spinal cord of DKO mice^10^. This finding is consistent with the lack of Cre expression in spinal cord for the human α-skeletal actin-Cre driver used to generate these mice (http://www.informatics.jax.org/recombinase/specificity?id=MGI:2447635&systemKey=4856356). The precise mechanism(s) responsible for denervation is unclear, and it is possible that different mechanisms operate on different fiber types depending on their activity level. Fiber loss, which occurred mainly between 3–6 weeks (Fig. 2d), and which appeared fiber-type unspecific (Supplementary Fig. 2), may have produced remodeling of motor unit size in the whole mutant SOL. Moreover, the longer open times of the γ-containing AChR^2^ might lead to increased local postsynaptic Ca^2+^, which might cause endplate degeneration and induction of slow-channel-like myasthenia^39^. Perhaps in the absence of ERK1/2, the tonic pattern of activity of type1 fibers and certain types of 2A fibers makes them more susceptible to synaptic Ca^2+^ overload than the phasic firing pattern of type 2B fibers. Alternatively or in addition, fiber-wide activity-dependent metabolic damage due to mitochondrial alterations or other general defects in post-weaning maturation may be responsible for the persistent denervation in young adult DKO SOL. Alterations in mitochondrial biogenesis and function were suggested by the reduction in COX and SDH activities and in mitochondrial markers, particularly PGC1-α (Fig. 6). Lack of PCG-1α reduces mitochondrial biogenesis^40^, but it also appears to modulate denervation-induced mitophagy, at least in fast-twitch muscles^41^. Further experiments are required to investigate a possible signaling link between ERK1/2 and PGC-1α, and its potential impact on mitochondrial dynamics. The mitochondrial alterations might trigger and/or increase the severity of denervation-induced wasting in ERK1/2-deficient SOL. To our knowledge, mitochondrial phenotypes have not been studied in *Chrne*^−/−^ mice. It will be interesting to do so to compare and contrast with our ERK1/2-deficient mice.

At 3 weeks we also saw a lack of 2B fibers in the DKO SOL (Fig. 2). These fibers were a small, very variable minority in control muscle at this age that in any event, were normally reduced to the levels present in DKO muscle by 5–6 weeks. Although this was an early event, it is unlikely that it is a major driver of the wasting of the DKO SOL as these fibers constitute such a minor component of the normal SOL. Furthermore, expression of *Myh4* mRNA (Supplementary Fig. 3) and 2B proportions by immunostaining, even in predominantly fast-twitch STN and TA muscles, are not affected by the lack of myofiber ERK1/2^10^.

Induction of AChRγ specifically in type 1 fibers is a common feature of many human neuromuscular disorders^42^. Although simple neurogenic denervation is the presumed main mechanism underlying this effect in most -but not all- cases, it is unclear why it is restricted to type 1 fibers. Given our results in mice, downregulation of myofiber ERK1/2 signaling might be an underappreciated mechanism driving this phenomenon.

## Materials and Methods

### Ethics Statement

Care and treatment of all animals followed the National Institutes of Health Guide for the Care and Use of Laboratory Animals, and were approved by the Institutional Animal Care and Use Committee of Texas A&M University under animal use protocols 2012-168 and 2014-060.

### Mice and Genotyping

Generation and genotyping of mice were described previously^10^. Cre was driven by the human α-skeletal muscle actin (*Hsa*) promoter. Mice deficient in germ line ERK1 and myofiber ERK2 came from the following cross: *Hsa-Cre*^+/−^*; Erk1*^+/−^*; Erk2*^*f/f*^ *x Hsa-Cre*^−/−^*; Erk1*^−/−^*; Erk2*^*f/f*^. The genotype of mutant animals (referred in the text as DKO mice) was *Hsa-Cre*^+/−^*; Erk1*^−/−^*; Erk2*^*f/f*^. The genotype of controls was *Hsa-Cre*^−/−^*; Erk1*^*+/?*^*; Erk2*^*f/f*^. Males and females of all genotypes were used in all experiments.

### Myofiber Morphological Analysis and Typing

Mice were euthanized by CO_2_ and muscles were immediately dissected. Muscles were embedded in tissue-tek (OCT, Sakura, Torrance, CA) within a plastic mold, frozen in liquid N_2_-cooled isopentane and stored at −80 °C until use. Dystrophin staining of transverse frozen sections was performed essentially as previously described except that sections were not fixed^43^. The same protocol was followed for laminin staining with a chicken polyclonal Ab IgY primary at 1:400, and a goat anti-chicken IgY H + L AlexaFluor 647 secondary at 1:500 (Abcam, Cambridge, MA). Fiber typing was done by multicolor immunofluorescence staining^16^ with some modifications. The same primary myosin heavy chain antibodies (Developmental Studies Hybridoma Bank, Iowa City, Iowa) at the previously reported dilutions were used to positively stain for type 1 (BA-F8), type 2A (SC-71), and type 2B (BF-F3). Type 2X fibers were those that remained unstained. Primary antibodies were visualized simultaneously with secondary antibodies (ThermoFisher Scientific/LifeTechnologies, Grand Island, New York) at 1:500 dilution: BA-F8 with Alexa-647 goat anti-mouse IgG2b; SC-71 with Alexa-488 goat anti-mouse IgG1; BF-F3 with Alexa-568 goat anti-mouse IgM. For fiber typing of individual cross sections, 10X images for each of the three fluorescence channels were taken with a Nikon wide-field E1000 microscope (Nikon Inc., Melville, New York), then merged and pseudocolored in Metamorph (Molecular Devices, Sunnyvale, California). To reconstruct an entire muscle cross section, the above overlapping images were assembled as a montage in Photoshop (Adobe Systems Inc., San Jose, CA). Individual fluorescence channels from these composites were counted to obtain the number of fibers of each type within one entire cross section. All fibers within the composites were counted. These numbers were used to derive the total number of fibers and relative fiber type composition. Fiber area was also derived from these images by first manually drawing a border around stained fibers within a channel using the trace region tool in Metamorph. These regions of interest were thresholded and analyzed with the integrated morphometric analysis tool to determine their area. As these images overlapped to cover the entire cross section, care was taken not to measure the same fibers more than once. At least 60% of fibers per muscle were processed this way for all but the 5–6 week controls, where at least 40% were so measured. Replicates per muscle per genotype were averaged for final quantification.

### AChRγ/ε Staining and Quantification

Rabbit antibodies to AChRγ and AChRε were a kind gift of Dr. Norihiro Yumoto (New York University) and were previously characterized^23^. Immunostaining of transverse serial sections was performed as described above with the following modifications: Blocking and antibody incubation for AChRγ experiments were performed in 10% normal goat serum in PBS + 0.1% Triton X-100, whereas for AChRε experiments they were blocked and incubated in 5% non-fat dry milk in PBS. These antibodies were visualized with rhodamine-goat anti-rabbit IgG (Jackson Immunoresearch Laboratories Inc., West Grove, PA) at 1:400. All AChRs were labeled with fluorescein-α-BTX, LifeTechnologies) at 1:3000. Nerve terminals were labeled with mouse monoclonal antibody to SV2 (Developmental Studies Hybridoma Bank) at 1:10, visualized with Alexa-647 goat anti-mouse IgG (LifeTechnologies) at 1:400. For quantification, 10 images per slide were taken for each separate channel per muscle at 20X magnification with a wide-field microscope (Nikon). BTX-positive synaptic sites were scored for AChR subunit and SV2 staining. Positive staining was that above extrasynaptic background on same fiber. Although we noticed differences in intensity of AChRγ/ε staining among synaptic sites, no efforts were made to quantify these levels separately. For AChRγ and type 1/2A MyHC co-staining, sections remained unfixed throughout. AChRγ, type 1 and type 2A MyHCs were visualized with the same secondary antibodies used above. Fluorescein-BTX was included to mark synaptic sites. Thus type 2A and BTX staining were both observed under fluorescein optics.

### Whole Mount Staining and Confocal Microscopy

Visualization of NMJs by whole mount and confocal microscopy was done as described previously^10^.

### Western Blotting

Muscle lysates were prepared in 25 mM Tris pH 7.4, 95 mM NaCl, 1 mM EDTA, 1 mM EGTA, 1% SDS, 10% Protease Inhibitor Cocktail (P8340, Sigma-Aldrich, St. Louis, MO), 5 mM NaF, 2 mM Na_3_VO_4_, 2.5 mM Na_4_P_2_O_7_. Protein analysis by western blotting was done as described previously^10^, except 30 μg of total lysate per sample was used. The antibody to PGC-1α (NBP1-48320. Novus Biologicals, Littleton, CO) was used at 1:2000. The antibody to SOD2 (SOD-111. Stressgen Biotechnologies, Victoria, BC) was used at 1:10000.

### *In Situ* Mitochondrial Enzyme Activity

Cytochrome oxidase (COX) and succinate dehydrogenase (SDH) activity on unfixed cross sections were performed as described^27^. Cross sections from control and DKO muscles were always run at the same time. Images were acquired with an EC3 camera and software (Leica Microsystems Inc., Buffalo Grove, IL), mounted on an Eclipse E1000 microscope (Nikon).

### Real-Time Quantitative PCR

Total RNA extraction, reverse transcription, and real-time PCR were performed essentially as previously reported^10^. 200 ng of total RNA per sample was used to generate cDNA. Cycle threshold (Ct) values obtained for 18 S rRNA were used to equalize differences in total RNA per sample. Transcript level fold-change was determined by the 2^−DeltaDeltaCt^ method^44^ and values were normalized to the Ct values obtained for control muscle for each gene. All Taqman primer sets and probes were from ThermoFisher/Life Technologies as follows: 18 S rRNA (4333760 F), *Atrogin1* (Mm00499523_m1), *Bnip3* (Mm01275600_g1), *Chrne* (Mm00437411_m1), *Chrng* (Mm00437419_m1), *Cytb* (Mm04225271_g1), *Drp1* (Mm01342903_m1), *Mfn1* (Mm00612599_m1), *Mfn2* (Mm00500120_m1), *Murf1* (Mm01185221_m1), *Musk* (Mm01346929_m1), *Myh1* (Mm01332489_m1), *Myh2* (Mm01332564_m1), *Myh3* (Mm01332463_m1), *Myh4* (Mm01332518_m1), *Myh7* (Mm01319006_g1), *Myog* (Mm00446194_m1), *Pgc-1α* (Mm01208835_m1), *Pgc-1β* (Mm00504720_m1), *Runx1* (Mm01213404_m1), *Sqstm1* (Mm00448091_m1), *Ucp3* (Mm00494077_m1).

### Statistical Analysis

All experiments were done at least in two biological replicas per genotype. Where biological replicas had a n ≤ 3 numerical data are expressed as mean ± SD. All real-time PCR experiments had at least 5 biological replicas per genotype. Numerical data for these experiments are expressed as mean ± SEM. Two-sample, two-tailed Student t-tests were used to compare means and were computed with Microsoft Excel (Microsoft Corporation, Redmond, WA). Wilcoxon rank sum test probabilities to compare distributions were computed at http://socr.stat.ucla.edu. Significance was set at P values of <0.05 for* and of <0.01 for**.

## Additional Information

**How to cite this article**: Wang, S. *et al*. Defective Acetylcholine Receptor Subunit Switch Precedes Atrophy of Slow-Twitch Skeletal Muscle Fibers Lacking ERK1/2 Kinases in Soleus Muscle. *Sci. Rep.* **6**, 38745; doi: 10.1038/srep38745 (2016).

**Publisher's note:** Springer Nature remains neutral with regard to jurisdictional claims in published maps and institutional affiliations.

## Supplementary Material

Supplementary Information
