# Corrigendum: Defective Acetylcholine Receptor Subunit Switch Precedes Atrophy of Slow-Twitch Skeletal Muscle Fibers Lacking ERK1/2 Kinases in Soleus Muscle

**DOI:** 10.1038/srep45420

**Published:** 2017-03-30

**Authors:** Shuo Wang, Bonnie Seaberg, Ximena Paez-Colasante, Mendell Rimer

Scientific Reports 6: Article number: 3874510.1038/srep38745; published online: 12 09 2016; updated: 03 30 2017

The Acknowledgements section in this Article is incomplete.

“This work was supported by National of Institute of Neurological Disorders and Stroke grant R21NS077177 and by Texas A&M Health Science Center funds to M.R. We thank N. Yumoto for AChR antibodies, J. Lawler for SOD2 antibodies, J. Samuel for access to a confocal microscope and R. Miranda for access to a real-time PCR machine. The MyHC and SV2 monoclonal antibodies developed by S. Schiaffino and K. Buckley were obtained from the Developmental Studies Hybridoma Bank developed under the auspices of the NICHD and maintained by The University of Iowa, Department of Biology, Iowa City, IA”.

should read:

“This work was supported by National Institute of Neurological Disorders and Stroke grant R21NS077177 and by Texas A&M Health Science Center funds to M.R. We thank N. Yumoto for AChR antibodies, J. Lawler for SOD2 antibodies, J. Samuel for access to a confocal microscope and R. Miranda for access to a real-time PCR machine. The MyHC and SV2 monoclonal antibodies developed by S. Schiaffino and K. Buckley were obtained from the Developmental Studies Hybridoma Bank developed under the auspices of the NICHD and maintained by The University of Iowa, Department of Biology, Iowa City, IA. The open access publishing fees for this article have been covered in part by the Texas A&M University Open Access to Knowledge Fund (OAKFund), supported by the University Libraries and the Office of the Vice President for Research”.

