# Supplementary material for: Defective Acetylcholine Receptor Subunit Switch Precedes Atrophy of Slow-Twitch Skeletal Muscle Fibers Lacking ERK1/2 Kinases in Soleus Muscle
Source: Sci Rep. 2016 Dec 9;6:38745. doi: 10.1038/srep38745 (PMC5146667; doi:10.1038/srep38745)
Supplement: Supplementary Information [file srep38745-s1.pdf]

Supplementary material for:

# "Defective Acetylcholine Receptor Subunit Switch Precedes Atrophy of Slow-Twitch Skeletal Muscle Fibers Lacking ERK1/2 Kinases in Soleus Muscle"

By: Shuo Wang, Bonnie Seaberg, Ximena Paez-Colasante and Mendell Rimer

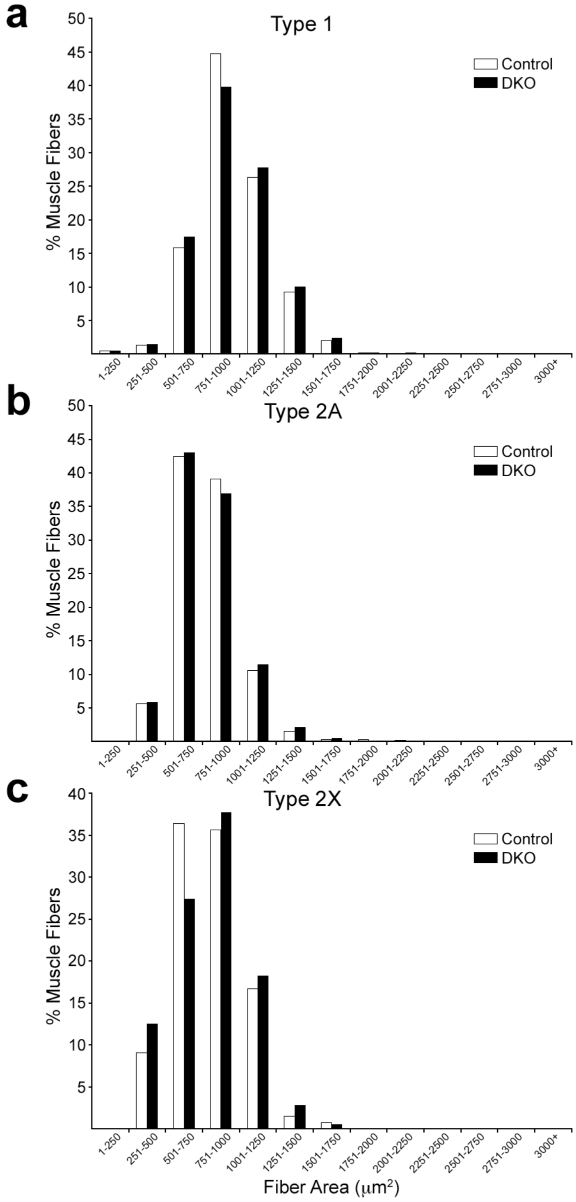


**Supplementary Figure 1. Distribution of fiber cross-sectional area by fiber type in the whole soleus muscle at 3 week of age.** Fiber area data for 3-week-old animals are grouped in 250 $\mu m^2$ bins along the X axis and the percentages of fibers in those bins are plotted on the Y axis. Fiber area distribution was similar between control and DKO muscles for all fiber types at this age. N=2 control and DKO male muscles. **(a)**

Type 1 fibers scored: 457 control, 417 DKO. **(b)** Type 2A fibers scored: 653 control, 802 DKO. **(c)** Type 2X fibers scored: 185 control, 175 DKO. No statistical difference was detected between control and DKO distributions by the Wilconox rank sum test.

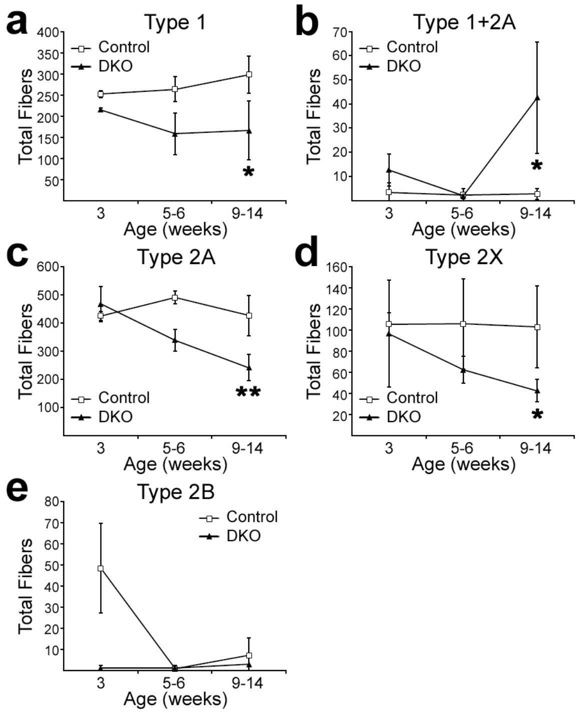


**Supplementary Figure 2. Fiber counts by fiber type at different postnatal times.** In young adult DKO SOL (9-14 weeks), type 1+2A hybrids were increased while type 1, 2A and 2X fibers were reduced relative to age-matched controls. Values are mean ± SD. N at 3 weeks: 3 Control, 3 DKO; n at 5-6 weeks: 3 Control, 2 DKO; n at 9 weeks: 2 Control, 2 DKO; n at 14 weeks: 2 control, 3 DKO. Data for 9 and 14 weeks were pooled. **, p<0.01; *, p<0.05; t-test v. control. P values: 0.013 **(a)**; 0.012 **(b)**; 0.002 **(c)**; 0.012 **(d)**.

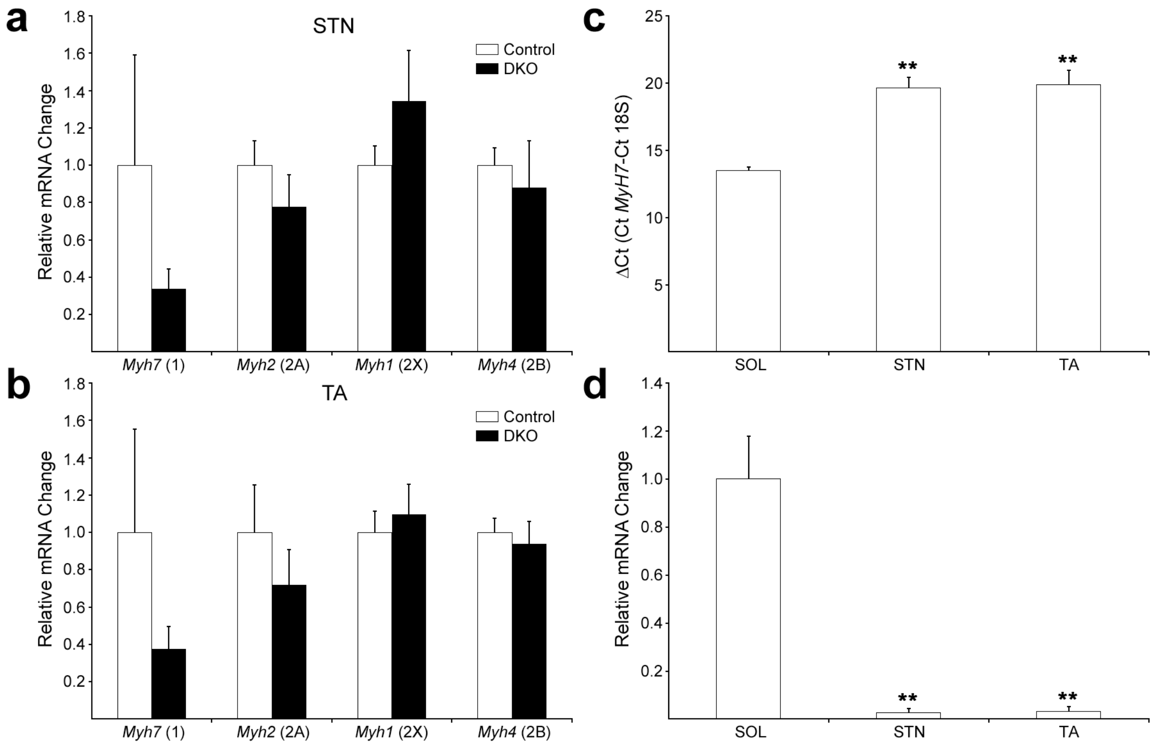


**Supplementary Figure 3. Relative *Myh* mRNA expression in young adult STN (a) and TA (b).** Real time PCR for MyHC genes at 9 weeks. N=6 per genotype/muscle. Values are mean + SEM. No statistically significant changes were detected. ***Myh7* mRNA levels in control SOL are ~30-fold higher than in either control TA or STN.** **(c)** ΔCt data ($Ct_{Myh7}$-$Ct_{18S}$) for control SOL, STN and TA. Note that the higher ΔCt the lower the expression level. **(d)** Normalized *Myh7* mRNA expression relative to levels in control SOL. N=6 per genotype/muscle. Values are mean + SEM. **, p<0.01; t-test vs. SOL.

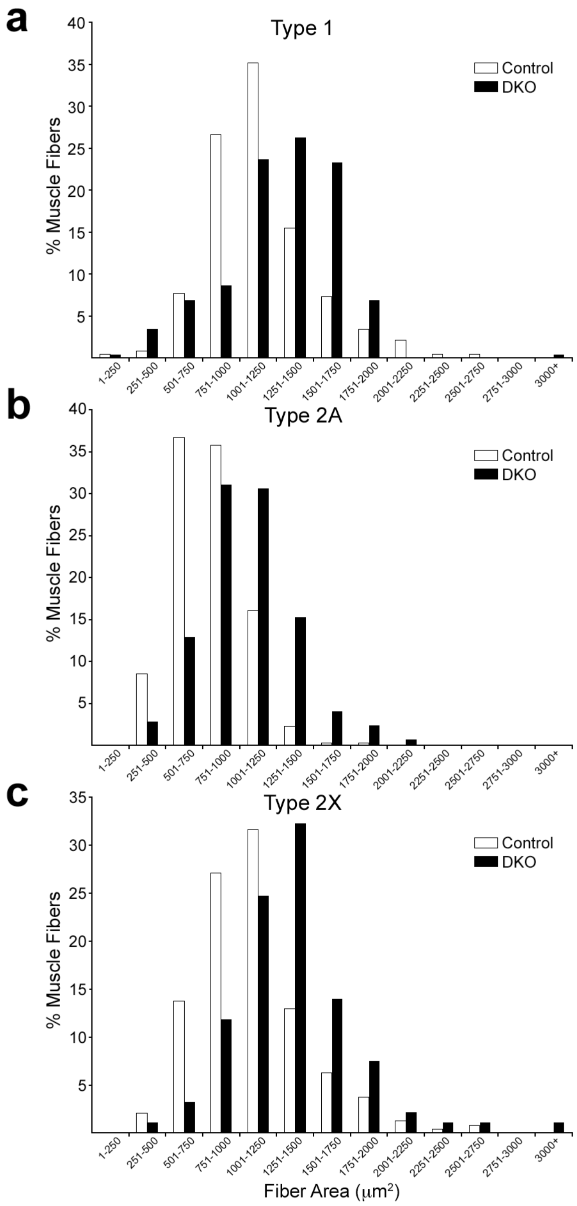


**Supplementary Figure 4. Distribution of fiber cross-sectional area by fiber type in the whole soleus muscle at 5-6 week of age.** Fiber area data for 5-6-week-old animals are grouped in 250 $\mu m^2$ bins along the X axis and the percentages of fibers in those bins are plotted on the Y axis. Fiber area shifted towards larger sizes for all fiber types at this age. N=2 control and DKO male muscles. **(a)** Type 1 fibers scored: 233 control, 233 DKO. **(b)** Type 2A fibers scored: 314 control, 418 DKO. **(c)** Type 2X fibers

scored: 240 control, 91 DKO. Distributions were compared statistically using the Wilconox rank sum test. $P < 2.04 \times 10^{-6}$ control v. DKO.

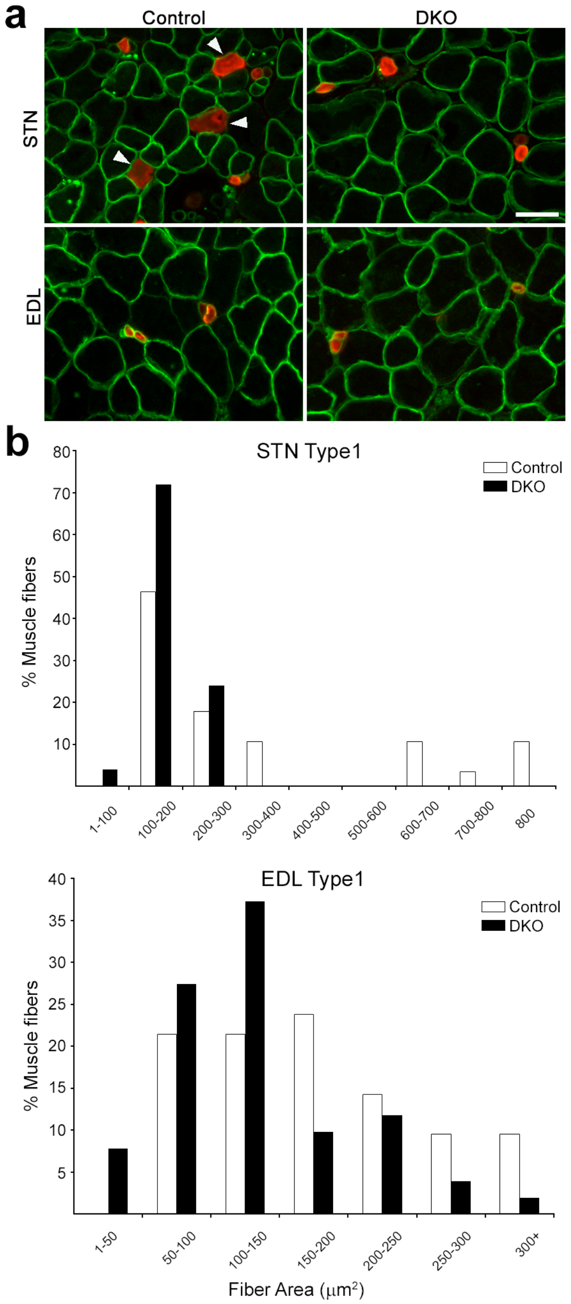


**Supplementary Figure 5. Type 1 fibers in DKO mice undergo atrophy in fast-twitch STN and EDL. (a)** Representative fields from cross sections of 14-week-old control and DKO STN and EDL stained for type 1 MyHC (red) and for laminin (green). Three, larger-area fibers in the control STN are indicated by arrowheads. Five smaller type 1 fibers are also visible in this field. EDL type 1 fibers are typically small regardless

of genotype. Scale bar: 50 μm. **(b)** Type 1 fiber area data were grouped in 100- (STN) and 50-$\mu m^2$ bins (EDL) along the X axes, respectively, and the percentages of fibers in those bins were plotted on the respective Y axes. In STN, fibers larger than 300 $\mu m^2$ were present in control but absent in the DKO. In EDL, fiber size distribution was slightly, but statistically significantly shifted to smaller sizes (p=0.004, t-test; p=0.008, Wilconox rank sum test). STN: n=3 muscles per genotype; 28 fibers, control; 25 fibers, DKO. EDL: n=4 muscles per genotype; 51 fibers, control; 42 fibers, DKO.

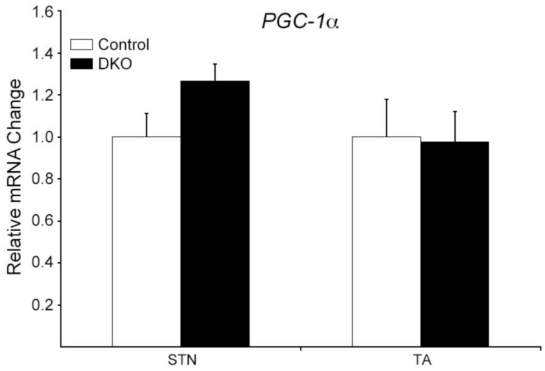


**Supplementary Figure 6. Relative *PGC-1α* mRNA expression in young adult STN and TA.** Real time PCR for *PGC-1α* at 9 weeks. N=6 per genotype/muscle. Values are mean + SEM.  No statistically significant changes were detected.

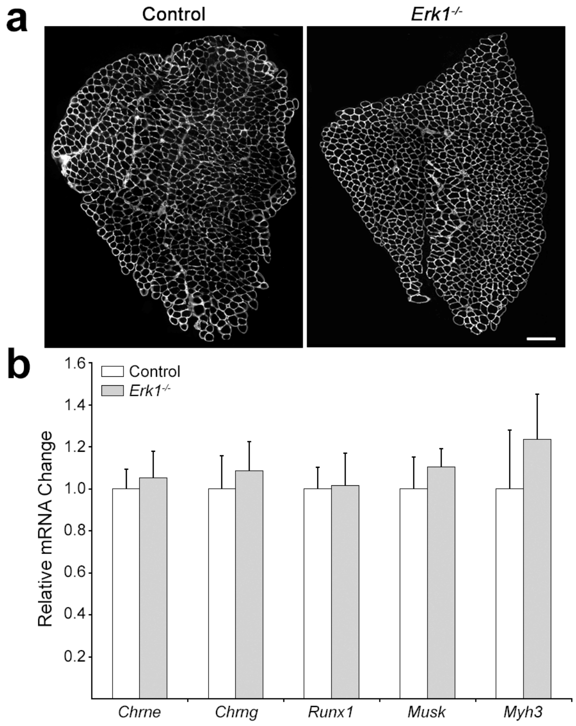


**Supplementary Figure 7. Morphological and molecular effects are absent in ERK1-deficient SOL. (a)** Cross sections of SOL from 9-week-old control and *Erk1*$^{-/-}$ stained for laminin to outline individual muscle fibers. Note overall similar morphology between mutant and control muscles. *Erk1*$^{-/-}$ SOL lacks the dramatic heterogeneity in fiber size found in DKO SOL (Fig 2a). Scale bar: 300 μm. **(b)** Real time PCR for *Chrne, Chrng, Runx1, Musk* and *Myh3* at 9 weeks for control and *Erk1*$^{-/-}$ SOL. N=6 for control and n=5 for *Erk1*$^{-/-}$. Values are mean + SEM. No statistically significant changes were detected.
